# Risk of Obstructive Sleep Apnea in Parkinson’s Disease: A Meta-Analysis

**DOI:** 10.1371/journal.pone.0082091

**Published:** 2013-12-09

**Authors:** Jun Zeng, Min Wei, Taoping Li, Wei Chen, Yuan Feng, Rong Shi, Yanbin Song, Wenling Zheng, Wenli Ma

**Affiliations:** 1 Institute of Genetic Engineering, Southern Medical University, Guangzhou, Guangdong, P. R. China; 2 Sleep Center, Nanfang Hospital, Southern Medical University, Guangzhou, Guangdong, P. R. China; 3 Department of Urology, Nanfang hospital, Southern Medical University, Guangzhou, Guangdong. P. R. China; The Chinese University of Hong Kong, Hong Kong

## Abstract

**Study Objectives:**

Sleep disorders are a common symptom of Parkinson’s disease (PD) and they significantly impair the sleep quality of the PD patients. However, there is no conclusive evidence to support the relation between PD and the prevalence of obstructive sleep apnea (OSA). The purpose of this meta-analysis review is to evaluate the association between PD and the prevalence of OSA.

**Methods:**

A comprehensive literature search was conducted on PubMed and Embase through July 2013. Only studies that referred to PD and the prevalence of OSA and that met the selection criteria were included in the analysis. The odds ratios (ORs) were used to evaluate the relationship of PD and the prevalence of OSA by the fixed-effect model.

**Results:**

Five eligible studies were analyzed in this study including 322 cases and 6,361 controls. The pooled-analysis showed the OR to be 0.60 (95% confidence interval (CI): 0.44 to 0.81, P = 0.001) and I^2^ = 0.0% (χ^2^ = 3.90, P = 0.420) in the fixed-effect model.

**Conclusions:**

Although we only included five small sample studies that indicated high homogeneity in the heterogeneity test, the results suggest that there is a significant negative association between PD and the prevalence of OSA; PD patients generally have a reduced prevalence of OSA. According to our analysis, these results are primarily due to the lower BMI of PD patients when compared with the general population controls.

## Introduction

Parkinson's disease (PD) is characterized by a group of conditions called motor system disorders which result from the loss of dopamine-producing brain cells. The four primary symptoms of PD include: trembling of hands, arms, legs, jaw, and face, rigidity or stiffness of the limbs and trunk, bradykinesia and postural instability or impaired balance and coordination. Early symptoms of PD are subtle and occur gradually. As the symptoms become more pronounced patients may have difficulties in walking, talking or performing other simple tasks. PD usually affects people over the age of fifty and it is a common disorder affecting approximately 300 per 100,000 people in industrialized countries [1-3].

Sleep disorders are reported in 74-98% of PD patients and sleep problems begin to occur in the early stage of the disease [4-11]. A patient with PD may suffer from different types of sleep disorders such as insufficient or fragmented sleep, sudden onset of sleep episodes, persistent excessive daytime sleepiness, rapid eye movement sleep behavior disorder, obstructive sleep apnea (OSA) and restless legs syndrome (RLS) [12]. Furthermore, recent studies of PD suggest high prevalence of excessive daytime sleepiness (EDS) and sleep disordered breathing (SDB). Although, EDS can result from coincidental OSA [13]. 

OSA affects at least 2% to 4% of the adult population and is increasingly recognized by the public [14]. Up to now, no well-designed study conclusively illustrates whether OSA occurs more frequent in PD patients than in the general population when both groups are matched for age, gender and race. Therefore, we performed a meta-analysis by analyzing relative studies to clarify the association between PD and the prevalence of OSA.

## Materials and Methods

### Identification and Eligibility of Relevant Studies

 A comprehensive literature search was performed, using the PubMed and Embase database, for relevant articles published up to July 2013 with the following key words; “Parkinson” or “Neurodegenerative disease*” or “Parkinson's disease”, combined with “sleep apnea”, “sleep apnoea”, “sleep disordered”, “sleep dysfunction” or SDB. The language filter was set to English. Articles from the Chinese national knowledge infrastructure (CNKI) database, which include articles mainly written in Chinese, were excluded due to the low quality of these studies. All selected studies were examined carefully and their bibliographies were checked for other relevant publications. In addition, we reviewed the references cited in the other relevant publications and identified additional articles missed by the database search.

### Inclusion and Exclusion Criteria

 The following inclusion criteria were used for study selection: (I) Human studies; (II) English language; (III) OSA diagnosed by polysomnography (PSG); (IV) use of case-control, cohort, cross-sectional or retrospective study design, (V) sufficient background data for estimating the OR with 95%CI of PD and non-PD patients. Exclusion criteria were (I) duplicate articles; (II) data from case report, reviews or letters to the editor; (III) subjects with ineligible general population controls (e.g. neurodegenerative control, close relative or spouses); (IV) criteria studies with patients that had neurodegenerative conditions, Parkinsonian syndromes, or Parkinsonism other than idiopathic PD.

### Data Extraction

 Information was carefully extracted from all eligible studies by two independent investigators according to the inclusion criteria listed above. Differences in the information extracted were resolved by consensus. For each study the characteristics collected include: First author’s name, year of publication, nationality, total number of cases and controls. Additional information of the tested group was collected which included gender, mean age and mean body mass index (BMI) for all PD patients and controls and PD duration and equivalent levodopa doses for PD patients. Because two studies [4,15] reported a non-integral number for OSA, we took the rounded number. We did not define any minimum number of patients, but the eligible general population control (e.g. no neurodegenerative control, close relative or spouses) was included in our meta-analysis. 

### Statistical Methods

 If the data was sufficiently similar and was presented as forest plots, meta-analysis was performed with STATA version 10.0 (STATA Corporation, College Station, TX, USA). The degree of correlation between PD and OSA risk was measured by ORs with 95%CIs. The random effects model was used to check the heterogeneity among studies. If I^2^ > 25%, the model would indicate a significant heterogeneity [16]. If I^2^ < 25%, which shows a minor heterogeneity or homogeneity, the fixed-effect model would be chosen for analysis [17,18]. In our research, a P value of > 0.10 for Q statistic indicated a lack of heterogeneity across the studies and therefore the fixed-effect model was used for analysis [19]. Furthermore, the risk of OSA associated with PD was estimated for each study.

### Methodological Quality Assessment

The Newcastle–Ottawa Scale was used to assess the methodological quality of the case–control or cohort studies. The Newcastle–Ottawa Scale contains eight items in total that are classified into three categories: selection (four items, a maximum of one star for each item), comparability (one item with a maximum score of two stars) and exposure/ outcome (three items, a maximum of one star for each item). A ‘‘star’’ represents a ‘‘high-quality’’ choice of the individual study [20]. Two reviewers, Wei Chen and Wenling Zheng, were blinded independently to assess the methodological quality without knowing each other’s assessing results, and any disagreement was settled by a discussion with a third reviewer, Yuan Feng.

## Results

### Characteristics of Included Studies

**Figure 1:**
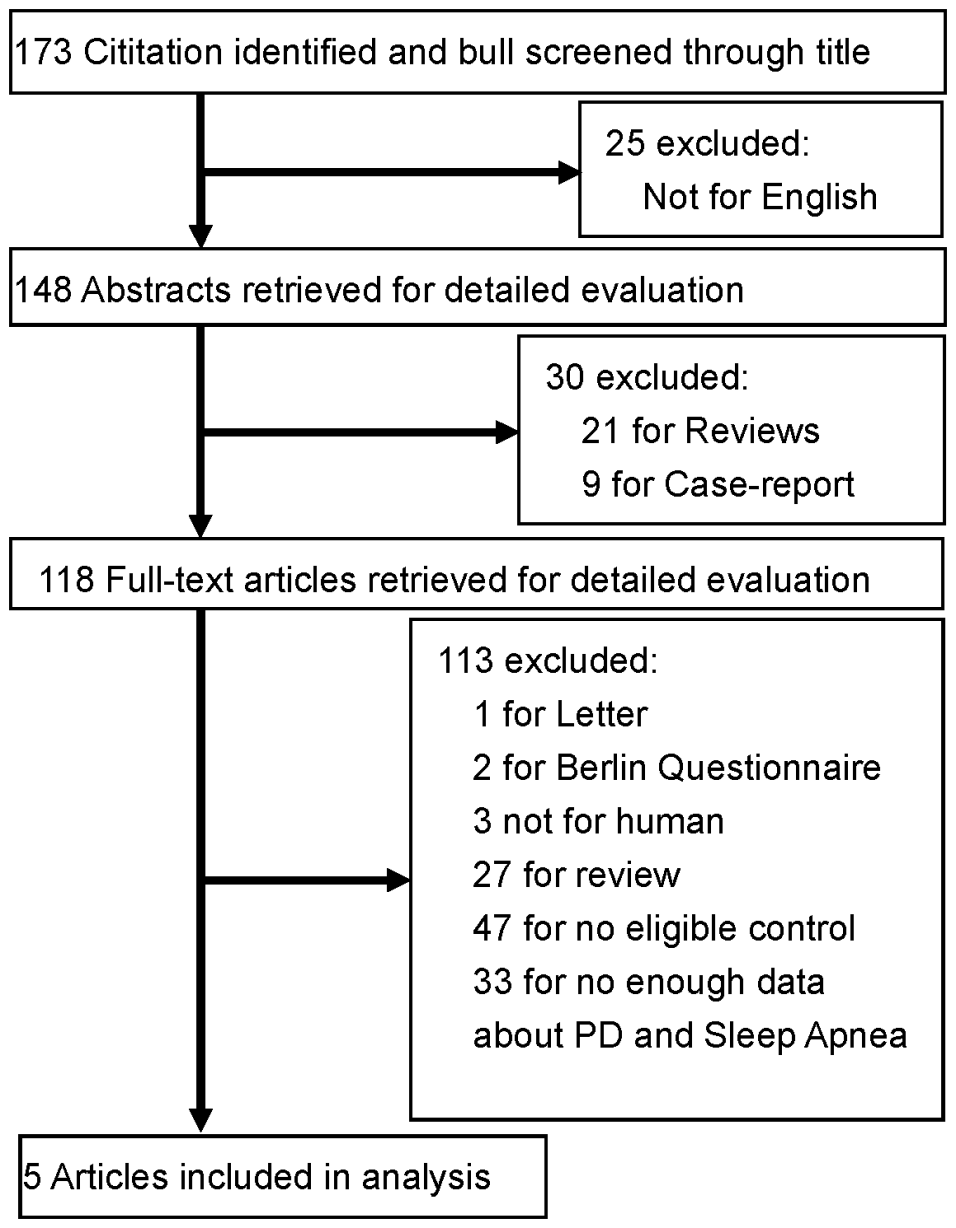
Flow chart for literature search and screening.

 The primary online database search identified 173 studies of potential interest. After reviewing their titles, we excluded 25 studies that were not in English and of the remaining 148 studies the abstracts were studied. Among them, 30 studies were excluded for not meeting inclusion criteria I and 118 full-text studies were finally reviewed. After the second selection round 113 studies were excluded because they did not meet the inclusion and exclusion criteria. For example, one study [21] was excluded concerning its ineligible control referring exclusion criteria V. Consequently, five studies were included in the meta-analysis for evaluating the association between PD and the risk of OSA [4,15,22-24]. Figure 1 shows the detailed procedure of the selection.

Overall, 322 cases and 6361 controls were included from five selected studies, 67.7% of the cases and 47.2% of the controls were male. According to previous reports, patients with OSA showed male predominance in the general population [25]. However, our study demonstrated that comorbidity of OSA had a male predominance in PD patients and a female predominance in the general population controls.

In the selected studies, the mean BMI was 24.92 in cases and 28.41 in controls, while the mean duration of PD was 7.04 years and the mean levodopa dose administered to PD patients was 530.45 mg/day. Based on the search results we established a database with the extracted information from each article (Table 1). The study designs were case-control or retrospective studies and OSA was diagnosed by PSG.

**Table 1:** Characteristics of eligible studies in the meta-analysis of PD and OSA risk.

**Author**		**Study**	**Selection**	**Sample. No**	**Male .No**	**Mean age**	**Mean BMI**	**PD**	**Mean Dose**	**Major Clinical Characteristics**
**(Year)**	**Nationality**	**Design**	**Case/Con**	**Case/Con**	**Case/Con (P)**	**Case/Con (Y)**	**Case/Con**	**Duration (Y)**	**LD (mg/day)**	**Sleep/PD**
**Diederich** (**2005**)[4]	Luxembourg	CC	Consecutive/Random	49/49	38 (77.6)/38 (77.6)	64.94/61.30	25.75/28.50	7.54	595.86	AHI, PLM, REM/NR
**Cohen de Cock** (**2010**)[22]	France	CC	Half-consecutive/NR	100/50	70 (70.0)/35 (70.0)	62.4/62.40	24.65/24.70	7.50	688.00	AHI, MMSE, ESS/NR
Trotti (2010)[24]	USA	RS	Convenient/NR	55/6132	37(67.3)/2848 (47.2)	63.90/62.90	26.80/28.50	5.80	331.00	AHI/NR
**Yong (2011**)[15]	Singapore	CC	Unselected/Unselected	56/68	34 (60.7)/38 (55.9)	65.40/59.30	22.80/23.90	6.40	409.40	AH, PLM, REM, RLS /UPDRS III, H & Y
Valko (2012)[23]	Switzerland	RS	Consecutive/Consecutive	62/62	39 (62.9)/43 (69.4)	58.44/58.19	24.95/27.69	7.59	510.90	AHI, ESS, EDS,PLM/UPDRS III, H & Y
**Total**				322/6361	218 (67.7)/3002 (47.2)	62.80/62.80	24.92/28.41	7.04	530.45	

**NR** *not reported*, **Case/Con** *case/control*, **BMI** *body mass index*, **LD** *levodopa*, P percentage of male in total, Y years, CC *case*-control, RS *retrospective*, **AHI** *apnea/hypopnea index*, **ESS** *Epworth sleepiness scale*, **PLM** *periodic leg movements*, **REM** *rapid eye movements*, **MMSE** *Mini-Mental State Examination*, **EDS** *Excessive daytime sleepiness*, **UPDRS III** *Unified Parkinson’s Disease Rating Scale* (*Part III = motor score*), **H &** Y Hoehn *& Yahr*

### Quality Assessment Results

 The Newcastle–Ottawa Scale scores for the three case-control studies ranged from 6 to 9, with a median of 7.67, which indicated that the case-control studies were of high quality (≥6). The median scores for the three categories were 3 for selection, 2 for comparability, and 2.67 for the ascertainment of exposure. The high scores in the Newcastle Ottawa Scale, including the almost full score in the comparability assessment, showed the high quality of the case-control studies (Table S1).

### Pooled-analysis Results

**Figure 2:**
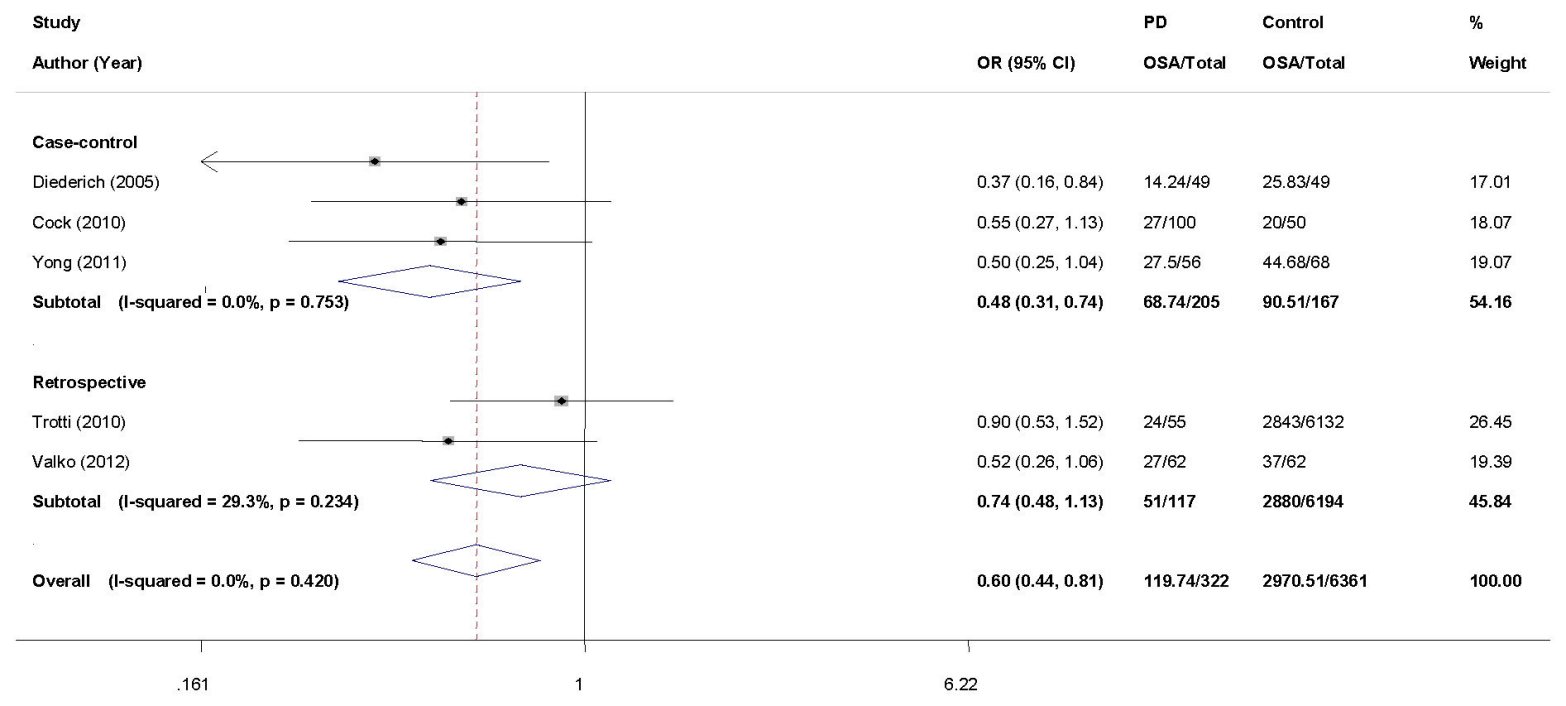
Forest plot for the risk of OSA in PD patients compared to controls. The squares and horizontal lines represent the study-specific OR and 95% CI (confidence interval). The area of the squares reflects the study specific weight (inverse of the variance). Diamond represents the pooled OR and 95% CI (fixed-effect model).

 Of the five studies included in the initial pooled-analysis, only one study [4] showed significant association between the risk of OSA and PD, with an OR of 0.37 (95%CI: 0.16 to 0.84). Therefore, we performed a meta-analysis to reveal whether the overall results could be different. Because the heterogeneity test showed that there was no heterogeneity among the individual studies (Heterogeneity test χ^2^ = 3.90, P = 0.420, I^2^ = 0.0%) we used a fixed-effects model for the pooled analysis. The analysis results showed a significant association between the decreased prevalence of OSA and idiopathic PD (OR = 0.60, 95%CI: 0.44 to 0.81) (Figure 2).

## Discussion

 In general, an increased frequency of OSA in PD patients is expected considering the known upper airway obstruction [26,27] and lung function abnormalities relative to controls populations. However, many studies showed similar rates of OSA in PD patients when compared to controls [4,15,22-24,28]. Some previous PSG studies illustrated that PD patients suffered from OSA and that the rates of patients suffering from moderate or severe OSA (AHI > 15) were more than 20% [4,22,29-31]. Meanwhile, another study found that severe OSA (AHI > 10) showed a higher prevalence (56%) in PD patients when compared to controls [32]. These results contradicted our analysis results, but it might be caused by the biased selection of the group. Some of these studies may have used patients who were selected for clinical purposes of PSG, EDS or other diagnosed criteria, so some biases might be allowed to overestimate the prevalence of OSA in PD. On the other hand, the result could be underestimated as well. For example, Cochen de Cock et al. [22] and her colleagues found a lower rate of OSA in PD patients than in controls by using hospitalized patients without PD as controls. According to the results, 21% of PD patients showed moderate or severe OSA (AHI ≥ 15) [22]. Taken together, these studies suggested that OSA was likely to be more prevalent in PD patients than in the general population. However, whether the prevalence of OSA in PD is lower than in the general population remains debatable. In our research, the results indicated: OR = 0.60 (95%CI: 0.44 to 0.81, P = 0.001) and I^2^ = 0.0% (χ^2^ = 3.90, P = 0.420) by fixed-effect model, indicating that all included studies were homogeneous. The results of our meta-analysis pooled-analysis showed that there was an association between PD and the decreased prevalence of OSA. The causes of this result will be discussed.

Previous reported risk factors for OSA include obesity, upper airway abnormalities, menopause, male gender, and age (the prevalence of OSA associated with a higher risk of mortality and morbidity increased with age and peaks at approximately 55 years old) [33]. Among all the factors, obesity is the strongest risk factor for OSA [34,35]. Hugo et al reported that the percentage of obesity in controls (79.7%) was significantly higher than in PD patients (66.1%) [36]. Another study showed that PD patients had a significantly lower BMI than the controls [37]. Each of the five selected studies showed a lower mean BMI in PD patients than in controls. Especially two studies [4,23] indicated a significant lower mean BMI in PD patients than in the controls. Since the results showed that obesity is the strongest risk factor for OSA and that PD patients appear relatively thinner, we suggest that the lower BMI of PD patients could be the major reason why PD patients showed lower prevalence of OSA.

Prior study revealed that a relatively high percentage of PD patients had upper airway obstruction (UAO), especially asymptomatic individuals [27]. However, upper airway abnormalities by themselves do not seem to be the cause of lower prevalence of OSA in PD patients. One case report showed that UAO could be reversed after the oral intake of levodopa [38] while other studies concluded that levodopa medication in patients with PD induced significant variations in UAO and PEF ratios. These research results suggest that levodopa might reverse UAO or OSA. However, further large-scale trials are necessary to evaluate the effect of levodopa on respiratory disorders related to UAO in patients with PD [39,40].

 Most studies showed that males were more likely to have PD [41-43] while in our selected studies, besides male predominance of PD patients, slight female predominance of controls was shown. Given the situation of female predominance in general population controls, the controls still showed higher risk of OSA. As male gender is one of the risk factors of OSA, male gender apparently could not be the cause of the lower prevalence of OSA in PD patients. Because all five studies were designed age-matched and showed similar mean age results, we tentatively took no account of the impact of age on the pooled results.

There are two main limitations to our study. First, all included studies are small sample studies, especially the PD patient number that is only 322. Therefore, more large-scale studies need to be conducted. Also, the number of the analyzed studies is limited, only studies reported in the English language were included, which could limit the generalizability of the analysis results of this study. Since only five studies were included in our analysis, it is clear that both statistical analysis and visual examination of funnel plots or other statistical tests (e.g. Egger’s test) have intensely restrained power which decreases with a reducing number of studies to test bias [44]. In order to avoid the occurrence of false positive results we fully reviewed the literature, set reasonable but strict inclusion and exclusion criteria and adjusted the level of significance (p > 0.10) to account for our results. However, we might still miss gray articles that we could not access.

## Conclusion

Our study suggests that PD patients have a lower risk of OSA when compared to the general population. Notably, the lower weight of PD patients seems to be the main protective factor against OSA. However, the results are partly limited by the small number of studies and further large-scale studies need to be conducted to verify our conclusion about the association between PD and the decreased prevalence of OSA.

## Supporting Information

Table S1 **Methodological quality of the included case-control studies.**
(DOCX)
