# Supplementary material for: Risk of Obstructive Sleep Apnea in Parkinson’s Disease: A Meta-Analysis
Source: PLoS One. 2013 Dec 9;8(12):e82091. doi: 10.1371/journal.pone.0082091 (PMC3857239; doi:10.1371/journal.pone.0082091)
Supplement: Table S1 — Methodological quality of the included case-control studies. (DOCX) [file pone.0082091.s001.docx]

**Table S1.** **Methodological quality of the included case-control studies.**

| Studies (Years) | **Selection** | **Comparability** | **Exposure** | **Total scores** |
| --- | --- | --- | --- | --- |
| Diederich (2005)[[4](#_ENREF_4)] | ☆☆ | ☆☆ | ☆☆ | 6 |
| Cock (2010)[[22](#_ENREF_22)] | ☆☆☆ | ☆☆ | ☆☆☆ | 8 |
| Yong (2011)[[21](#_ENREF_21)] | ☆☆☆☆ | ☆☆ | ☆☆☆ | 9 |
